# Differential Phosphorus Acquisition Strategies and Adaptive Mechanisms Evolved by Three *Lespedeza* Species to Tackle Phosphorus Deficiency

**DOI:** 10.3390/plants14203124

**Published:** 2025-10-10

**Authors:** Jingchong Li, Hao Shi, Guanqiang Zuo, Shasha Li, Yafei Chen, Shiwen Wang

**Affiliations:** 1 State Key Laboratory of Soil and Water Conservation and Desertification Control, the Research Center of Soil and Water Conservation and Ecological Environment, Chinese Academy of Sciences and Ministry of Education, Yangling 712100, China; ljc_123mail@163.com; 2 Institute of Soil and Water Conservation, Chinese Academy of Sciences and Ministry of Water Resources, Yangling 712100, China; 3 University of Chinese Academy of Sciences, Beijing 100049, China; 4 College of Soil and Water Conservation Science and Engineering, Northwest A&F University, Yangling 712100, China; shzyy@nwafu.edu.cn (H.S.); byndzgq@163.com (G.Z.); lishasha@nwafu.edu.cn (S.L.); cyf05270924@163.com (Y.C.)

**Keywords:** phosphorus deficiency, absorption kinetics, phosphatases, root exudation

## Abstract

Phosphorus (P) is essential for plant growth but is frequently limited in soils. *Lespedeza* species are well-known for their ecological and economic benefits, as well as their tolerance to nutrient-poor soils. This study investigated the P acquisition strategies and adaptive mechanisms of three *Lespedeza* species (*L. davurica*, *L. bicolor*, and *L. cuneata*), focusing on biomass allocation, P distribution, root exudation, and absorption kinetics under P deficiency. Under P deficiency, *L. davurica* and *L. bicolor* allocated more biomass to roots to enhance P acquisition, whereas *L. cuneata* increased specific root length and area. Moreover, all three species preferentially allocated P to roots, but *L. bicolor* showed higher P content in stems and leaves than the others. P absorption kinetics indicated that Michaelis constant (*K_m_*) and equilibrium concentration (*C_min_*) were significantly decreased in all three species under P deficiency, with *L. bicolor* exhibiting the strongest P affinity and acquisition capacity. Secretion analysis revealed that while *L. davurica* and *L. cuneata* secreted higher levels of organic acids under P deficiency, exudates from *L. bicolor* were significantly enriched in acid phosphatase activity. Overall, the three *Lespedeza* species developed distinct P acquisition and adaptive strategies to cope with P deficiency, with *L. bicolor* demonstrating the greatest low-P tolerance and most efficient adaptive mechanisms.

## 1. Introduction

Phosphorus (P) is an essential nutrient for plants, playing crucial roles in energy metabolism, signal transduction, photosynthesis, and the biosynthesis of nucleic acids and fatty acids [1,2,3]. However, due to anthropogenic activities, climate change, land-use patterns, and other factors, the availability of P in global soils is significantly deficient [4,5]. P has become a major limiting factor for plant growth in grasslands, forests, and agricultural ecosystems [5,6,7]. Furthermore, the availability of soil nutrients may further decrease with global warming [8,9]. Although P fertilizers can mitigate low soil P availability, the primary source, phosphate rock, is finite and non-renewable [10]. Enhancing plant P use efficiency has thus become crucial for future sustainability, necessitating a deeper understanding of P sensing and response mechanisms in plants.

Plants employ various morphological strategies to alleviate P deficiency, such as increasing root surface area, specific root length, and lateral root density [11,12,13,14]. These adaptations involve changes in root architecture and the formation of cluster roots, which increase the contact area between roots and soil, thereby promoting P absorption [14,15,16]. Root morphological responses to P deficiency have been extensively documented in various plant species, including grains, rapeseed, legumes, and perennial pasture grasses [13,16,17,18,19]. Notably, root plasticity varies across plant species and genotypic background, suggesting that plants have the potential for selective breeding or evolutionary adaptation to improve survival and growth under P-limiting conditions [14,20].

In addition to modulating root morphology to cope with P deficiency, plants can also adjust root physiology [11]. Roots release various exudates, including phosphatases, organic acids, and phytases [12,19], which facilitate the mobilization and desorption of P from soil particles, thus improving soil P availability. For instance, the exudation of organic acid carboxylates improves soil P solubility and uptake, particularly in soils with high P fixation or deficiency [11]. Rather than acidifying the rhizosphere, these carboxylates enhance P mobilization mainly through ligand exchange and chelation reactions, which displace phosphate from Fe/Al oxides and humic surfaces, thereby increasing P availability in the soil solution [21,22]. In addition to root structural changes and increased exudate production, root nutrient uptake kinetics are crucial for fully understanding plant responses to P deficiency [23,24]. This approach provides an effective way to identify plants with efficient P absorption.

*Lespedeza* is a group of perennial leguminous plants that are highly valued for their forage, medicinal, and ecological benefits. They enhance soil fertility, prevent soil erosion, and exhibit tolerance to poor soil conditions such as acidity, drought, infertility, and P deficiency [25,26]. Previous studies have demonstrated that different *Lespedeza* species exhibit varying strategies for coping with P deficiency. For example, *L. cuneata* has been shown to modulate root morphology as an adaptive strategy to P deficiency, while *L. bicolor* secretes organic acids like citric and malic acids to enhance P availability [26]. Furthermore, the *Nguyenibacter* sp. L1 strain, isolated from the rhizosphere of *L. bicolor*, can dissolve aluminum phosphate by secreting gluconic acid, thus alleviating P deficiency [27]. In addition, *L. davurica* has been found to optimize nitrogen and P uptake when intercropped with other plants under limited resource conditions [28]. Despite these insights, a comparative study of the specific mechanisms and the differences in P tolerance among *Lespedeza* species is still lacking.

This study compares the P adaptive strategies of three *Lespedeza* species—*L. davurica*, *L. bicolor*, and *L. cuneata*. Although studies have shown that these species exhibit tolerance to P deficiency, their precise differences in P tolerance and response mechanisms remain unclear. Based on available research, we hypothesize that these species adopt different strategies to cope with P deficiency. These strategies may involve changes in root morphology, organic acid secretion, increased phosphatase activity, and improved P absorption efficiency. Specifically, *L. bicolor* may enhance P absorption efficiency through increased phosphatase activity. In contrast, *L. davurica* is expected to allocate more carbon to root growth, while *L. cuneata* may modify its root morphology to improve P acquisition. Additionally, these species may increase organic acid secretion to mobilize P from the soil. To explore these hypotheses, we compared their biomass allocation, P distribution, root exudate profiles, and P absorption kinetics under P-sufficient and P-deficient conditions. Additionally, we have investigated their P acquisition strategies and elucidated how these strategies influence their adaptability to P deficiency. By identifying and distinguishing *Lespedeza* species with varying P adaptation trade-offs, this study aims to elucidate their mechanisms for low-P tolerance and provide a theoretical framework for improving P availability in these plants.

## 2. Results

### 2.1. Biomass Allocation and Root Morphological Characteristics

**Figure 1:**
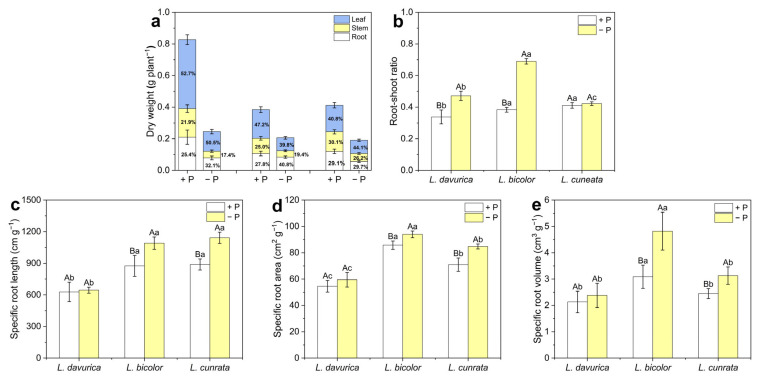
Biomass allocation and root morphological characteristics of three *Lespedeza* species at 21 days after transfer (DAT) to hydroponics under different P levels. (**a**) Dry weight of different tissues. (**b**) Root-shoot ratio. (**c**) Specific root length. (**d**) Specific root area. (**e**) Specific root volume. −P: P-deficient condition, +P: P-sufficient condition. Data are shown as Mean ± SD (*n* = 5). One-way ANOVA was performed to compare differences at different P levels for the same species, and between species at the same P level, with post hoc Duncan’s multiple range test. Different uppercase letters in figures indicate significant differences at different P levels for the same species (*p* < 0.05), and different lowercase letters in figures indicate significant differences between species at the same P levels (*p* < 0.05).

The biomass allocation and root morphological characteristics of the three *Lespedeza* species were analyzed under two P levels. Under P sufficiency, *L. davurica* exhibited the highest biomass at 0.83 g plant^−1^, while *L. bicolor* (0.38 g plant^−1^) and *L. cuneata* (0.41 g plant^−1^) showed no significant biomass difference (Figure 1a). Under P deficiency, biomass decreased significantly in all species, with *L. davurica* showing the greatest reduction (70.3%) and *L. bicolor* the least (46.4%) (Figure 1a).

Under P deficiency, the root biomass proportion of *L. davurica* and *L. bicolor* increased to 32.1% and 40.8%, respectively, compared to 25.4% and 27.8% under P sufficiency (Figure 1a). Their root-to-shoot ratios also increased, indicating enhanced carbon allocation (Figure 1b). Root morphological analysis revealed that specific root length (*p* = 0.002), specific root area (*p* = 0.034), and specific root volume (*p* = 0.001) of *L. bicolor* increased significantly under P deficiency, while *L. davurica* showed no such changes (Figure 1c–e). Notably, *L. cuneata* showed no significant change (*p* = 0.588) in the root biomass proportion and root-to-shoot ratio under P deficiency, but its specific root length, specific root area, and specific root volume increased markedly (Figure 1b–e). Additionally, under P sufficiency, root biomass proportions were similar across the three species: *L. davurica* (25.4%), *L. bicolor* (27.8%), and *L. cuneata* (29.1%). However, under P deficiency, *L. bicolor* had the highest root biomass proportion (40.8%) (Figure 1a), suggesting that *L. bicolor* allocated more carbon to its roots than the other two species.

### 2.2. Growth Analysis

To assess the growth characteristics of the three *Lespedeza* species under two P levels, we calculated their RGR and several leaf area-related parameters.

**Figure 2:**
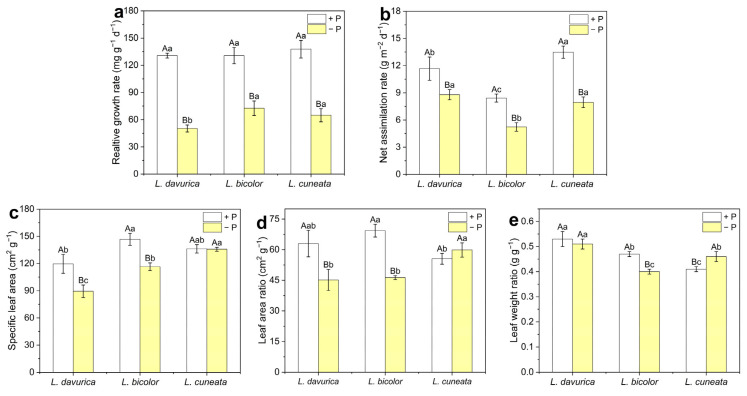
Parameters of growth analysis of three *Lespedeza* species under different P levels. (**a**) Relative growth rate (RGR). (**b**) Net assimilation rate (NAR). (**c**) Specific leaf area (SLA). (**d**) Leaf area ratio (LAR). (**e**) Leaf weight ratio (LWR). −P: P-deficient condition, +P: P-sufficient condition. Data are shown as Mean ± SD (*n* = 5). One-way ANOVA was performed to compare differences at different P levels for the same species, and between species at the same P level, with post hoc Duncan’s multiple range test. Different uppercase letters in figures indicate significant differences at different P levels for the same species (*p* < 0.05), and different lowercase letters in figures indicate significant differences between species at the same P levels (*p* < 0.05).

Under P sufficiency, no significant differences (*p* > 0.099) in RGR were observed among the three species (Figure 2a). Under P deficiency, the RGR of all species decreased significantly (*p* = 0.000 *) compared to the P sufficiency, with *L. davurica* showing the greatest reduction (50.30 mg g^−1^ d^−1^) and *L. bicolor* the least (72.64 mg g^−1^ d^−1^). Notably, *L. bicolor* had a significantly higher RGR than *L. davurica* (*p* = 0.000 *) and *L. cuneata* (*p* = 0.003) under P deficiency (Figure 2a), suggesting better adaptation to P deficiency.

Under P sufficiency, *L. bicolor* exhibited the highest SLA, while *L. davurica* had the lowest SLA (Figure 2c). Under P deficiency, the SLA of *L. davurica* (*p* = 0.002) and *L. bicolor* (*p* = 0.002) decreased significantly, whereas that of *L. cuneata* remained unchanged, resulting in the highest (*p* = 0.031) SLA for *L. cuneata*. Under P sufficiency, *L. cuneata* exhibited the lowest LAR (Figure 2d). However, under P deficiency, the LAR of *L. cuneata* was 32.6% and 29.3% higher than that of *L. davurica* and *L. bicolor*, respectively. *L. davurica* exhibited the highest LWR under both P-sufficient and P-deficient conditions. Moreover, under P deficiency, the LWR of *L. davurica* remained stable, while that of *L. bicolor* decreased (*p* = 0.000 *) and that of *L. cuneata* increased (*p* = 0.018) (Figure 2e).

### 2.3. Phosphorus Allocation Patterns

**Figure 3:**
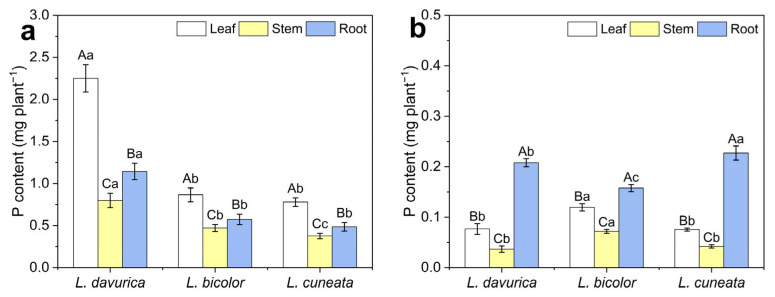
The P contents of leaves, stems, and roots in three *Lespedeza* species at 21 DAT under different P levels. (**a**) P-sufficient condition. (**b**) P-deficient condition. Data are shown as Mean ± SD (*n* = 3). One-way ANOVA was performed to compare P content differences in different tissues for the same species at the same P level, and between species for the same tissue, with post hoc Duncan’s multiple range test. Different uppercase letters in figures indicate significant differences between different tissues for the same species at the same P level, and different lowercase letters in figures indicate significant differences between species for the same tissue at the same P level.

To clarify the P allocation patterns of the three *Lespedeza* species, the P content of various tissues was analyzed under two P levels. Under P deficiency, all three species allocated more P to root tissues, with P concentration highest in roots and lowest in stems (Figure 3b). Interestingly, although the roots of *L. bicolor* had the lowest P content, its leaves and stems with higher P content than *L. davurica* (*p* = 0.000 *) and *L. cuneata* (*p* = 0.000 *) (Figure 3b).

Under P sufficiency, all three species showed a P partitioning pattern of leaf > root > stem (Figure 3). Notably, although *L. davurica* had the highest total P, attributed to its large dry matter accumulation, the P concentrations in the leaf and stem tissues of *L. bicolor* were significantly higher than those in *L. davurica* (*p* = 0.000 *) and *L. cuneata* (*p* = 0.000 *) (Figure S1b).

### 2.4. Phosphorus Absorption Kinetics

**Figure 4:**
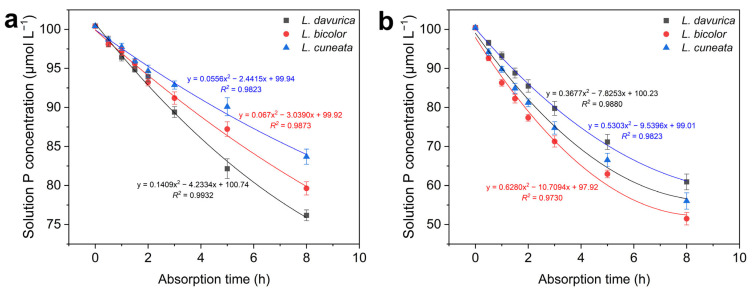
Absorption kinetic curve in three *Lespedeza* species at 21 DAT under different P levels. (**a**): P-sufficient condition, (**b**): P-deficient condition. Data are shown as Mean ± SD (*n* = 3).

**Figure 5:**
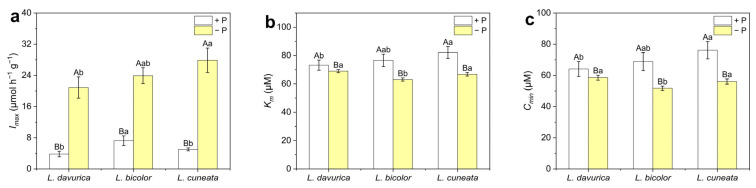
Parameters of the absorption kinetics of three *Lespedeza* species under different P levels. (**a**) Maximum potential for plant P absorption; (**b**) Michaelis constant; (**c**) Equilibrium concentration. −P: P-deficient condition, +P: P-sufficient condition. Data are shown as Mean ± SD (*n* = 3). One-way ANOVA was performed to compare differences at different P levels for the same species, and between species at the same P level, with post hoc Duncan’s multiple range test. Different uppercase letters in figures indicate significant differences at different P levels for the same species (*p* < 0.05), and different lowercase letters in figures indicate significant differences between species at the same P levels (*p* < 0.05).

For a quantitative assessment of P absorption under both P-sufficient and P-deficient conditions, the P absorption curves depicted in Figure 4 were constructed for each of the *Lespedeza* species and were used to calculate kinetic absorption parameters (Figure 5). All three species exhibited significantly enhanced P absorption rates and capacities under P deficiency, with *L. bicolor* demonstrating a more pronounced boost in P absorption compared with the other two species (Figure 4).

Specifically, three kinetic parameters—*I_max_*, *K_m_*, and *C_min_*—were significantly changed (*p* = 0.000 *) under P deficiency in three *Lespedeza* species (Figure 5). *I_max_* reflects the maximum P absorption potential, *K_m_* indicates the species’ affinity for P under low availability, and *C_min_* represents the P concentration at which absorption begins. Under P deficiency, all species increased their *I_max_* and decreased *K_m_* and *C_min_*, with *L. bicolor* exhibiting the most pronounced changes (Figure 5). In addition, under P sufficiency, *L. bicolor* showed the highest *I_max_*, while *L. davurica* had the lowest *K_m_* and *C_min_*. These results suggest that the three *Lespedeza* species employ different strategies to adapt to varying P availability, with enhancing their P absorption capacity and affinity being key mechanisms for responding to P deficiency.

### 2.5. Acid Phosphatase Activity and Organic Acid Exudation

**Figure 6:**
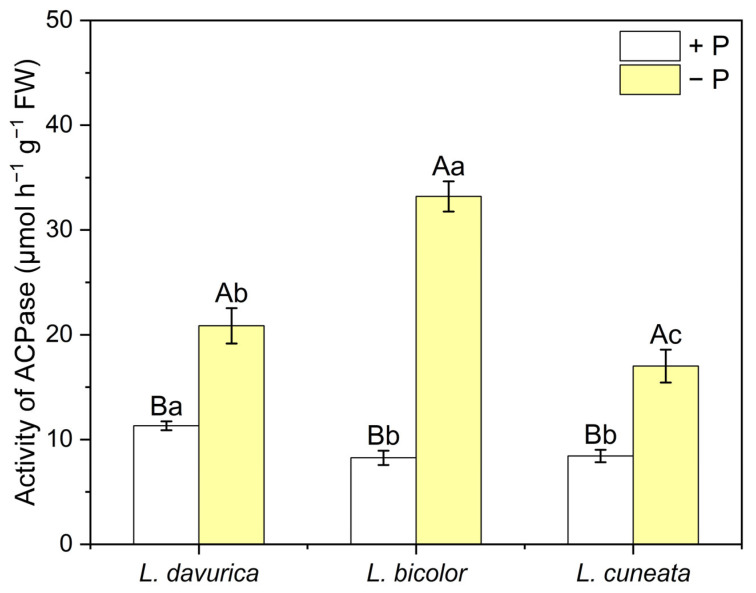
Activity of acid phosphatase (ACPase) in three *Lespedeza* species at 21 DAT under different P levels. +P: P-sufficient condition, −P: P-deficient condition. Data are shown as Mean ± SD (*n* = 3). One-way ANOVA was performed to compare differences at different P levels for the same species, and between species at the same P level, with post hoc Duncan’s multiple range test. Different uppercase letters in figures indicate significant differences at different P levels for the same species (*p* < 0.05), and different lowercase letters in figures indicate significant differences between species at the same P levels (*p* < 0.05).

The acid phosphatase activity in root exudates offers valuable insights into how plants respond to varying P availability. In this study, we compared the acid phosphatase activity in root exudates of three *Lespedeza* species under two P levels. Under P sufficiency, *L. davurica* exhibited significantly higher acid phosphatase activity than *L. bicolor* (*p* = 0.015) and *L. cuneata* (*p* = 0.008), with no significant difference observed between the latter two species (*p* = 1.000) (Figure 6). Under P deficiency, the acid phosphatase activity of *L. davurica*, *L. bicolor*, and *L. cuneata* increased by 84.3%, 302.4%, and 101.8%, respectively, compared to P sufficiency. Moreover, under P deficiency, acid phosphatase activity is highest in *L. bicolor* and lowest in *L. cuneata* (Figure 6).

**Figure 7:**
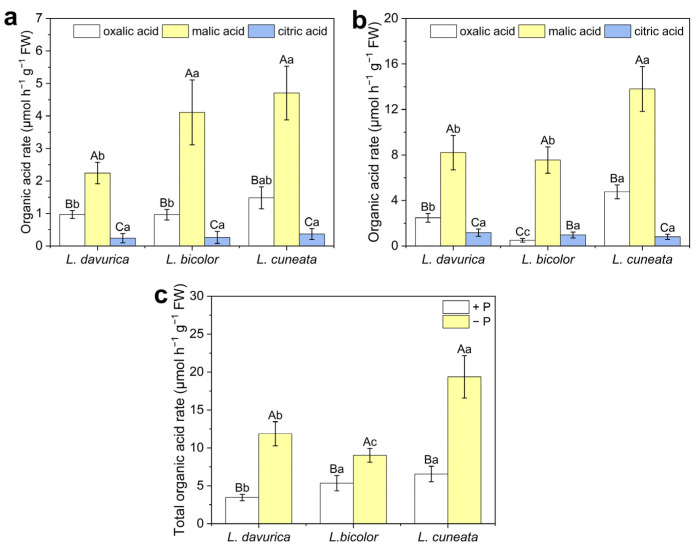
Exudation of organic acids from three *Lespedeza* species at 21 DAT under different P levels. (**a**): P-sufficient condition, (**b**): P-deficient condition. (**c**): Total organic acid (oxalic acid + malic acid + citric acid) secretion rate under two P conditions. +P: P-sufficient condition, −P: P-deficient condition. Data are shown as Mean ± SD (*n* = 3). One-way ANOVA was performed to compare differences in organic acid content within the same species at the same P level, differences between species for the same organic acid, as well as differences in total organic acid content within the same species at different P levels and between species at the same P level, followed by post hoc Duncan’s multiple range test. In figures (**a**,**b**), different uppercase letters indicate significant differences in organic acid content within the same species at the same P level (*p* < 0.05), and different lowercase letters indicate significant differences between species for the same organic acid at the same P level (*p* < 0.05). In the figure (**c**), uppercase letters indicate significant differences in total organic acid content within the same species at different P levels (*p* < 0.05), and lowercase letters indicate significant differences between species at the same P level (*p* < 0.05).

We also measured the content of oxalic acid, malic acid, and citric acid in root exudates. Under P sufficiency, all three species exhibited high malic acid secretion, with *L. davurica* showing a significantly lower secretion rate compared to *L. bicolor* (*p* = 0.000 *) and *L. cuneata* (*p* = 0.000 *) (Figure 7a). Under P deficiency, the organic acid secretion rate of all three *Lespedeza* species increased significantly, with the most pronounced increase in malic acid observed for *L. bicolor*. Additionally, *L. cuneata* exhibited the highest secretion rates of both malic acid (*p* = 0.002 *) and oxalic acid (*p* = 0.000 *) (Figure 7b). The total organic acid secretion rate showed that, regardless of P availability, *L. cuneata* exhibited a stronger organic acid secretion capacity than *L. davurica* (*p* = 0.000 *) and *L. bicolor* (*p* = 0.000 *) (Figure 7c).

## 3. Discussion

*Lespedeza* is a group of perennial leguminous plants highly valued for their forage, medicinal, and ecological benefits, as well as their strong tolerance to drought and poor soil conditions. In this study, we compared three *Lespedeza* species (*L. davurica*, *L. bicolor*, and *L. cuneata*) under P-sufficient and P-deficient conditions, analyzing their biomass allocation, P allocation, P absorption kinetics, root exudate characteristics, and P acquisition strategies. The results showed that *L. bicolor* exhibits the greatest tolerance to low P, as it shows the smallest reduction in RGR and has the lowest *C_min_*. Additionally, root secretion analysis reveals that *L. davurica* and *L. cuneata* secrete more organic acids under P stress, while *L. bicolor* demonstrates higher acid phosphatase activity. The findings revealed that these species have developed different P acquisition and adaptive strategies to tackle P deficiency.

Plant biomass allocation is a key response to P deficiency [24]. Under P deficiency, *L. davurica* and *L. bicolor* exhibited reduced shoot growth and a higher root-shoot ratio (Figure 1). This ratio is an important indicator of how plants partition photosynthates between shoots and roots. Under P deficiency, allocating more photosynthetic carbon to roots can promote root growth or enhance exudation, both of which facilitate P acquisition [29,30]. A previous study showed that sucrose-mediated gene expression (*SUCs* and *SWEETs*) was significantly upregulated under P deficiency, which may promote root growth and explain the increased root-shoot ratio [31]. However, *L. cuneata* did not follow this strategy, and did not show a significant increase in root-shoot ratio under P deficiency, despite significant inhibition of shoot growth (*p* = 0.000 *) (Figure 1). Some researchers indicate that shoot growth inhibition is less pronounced under mild P deficiency, as increased root growth can offset the adverse effects on the shoots [32]. The significant reduction in biomass accumulation in *L. cuneata* suggests severe P limitation. We hypothesize that this could reflect a trade-off mechanism, where *L. cuneata* may prefer to maintain growth by balancing limited photosynthetic carbon and P, rather than allocating more carbon to roots for P acquisition. Alternatively, the increase in P acquisition may not depend heavily on the extensive allocation of photosynthetic carbon to the roots. This is supported by our observation of enhanced P acquisition ability in *L. cuneata* under P deficiency conditions (Figure 5).

In addition to allocating more carbon to roots to promote root growth, root architecture and morphology are also crucial for P acquisition [14]. These traits are species- and genotype-specific and are influenced by P availability [13]. In this study, *L. bicolor* and *L. cuneata* showed significantly higher specific root length (*p* < 0.001) and specific root area (*p* < 0.006) than *L. davurica* under both P levels (Figure 1c,d). This difference may be attributed to variations in root morphology among the species. *L. davurica* has a prominent taproot with fewer lateral roots, while *L. bicolor* and *L. cuneata* have more lateral roots, resulting in higher specific root length and specific root area (Figure S3). Plants typically respond to P deficiency by increasing root length and decreasing root diameter, changes that increase specific root length and specific root area [13,15,18]. These changes suggest that plants can improve nutrient uptake efficiency by increasing root extension per unit biomass or promoting root branching to access a larger soil volume. This strategy reduces root construction costs and stimulates fine root development under low-P stress, serving as a resource-acquisition approach [33,34]. Both *L. bicolor* and *L. cuneata* seem to employ this strategy to enhance P acquisition. The more pronounced reductions in *K_m_* and *C_min_* values in *L. bicolor* and *L. cuneata* under P deficiency, compared to *L. davurica*, also support this viewpoint (Figure 5). However, no significant increase in specific root length (*p* = 0.768) or specific root area (*p* = 0.342) was observed in *L. davurica* under P deficiency (Figure 1c,d). Instead, *L. davurica* exhibited lower lateral root density and slower growth, although its primary root length increased significantly (Figure S3). This suggests that *L. davurica* may have adopted a resource-conservative strategy, possibly evolved due to its long-term residence in a semi-arid region, where it has an advantage in soils with limited water and nutrients. This strategy allows *L. davurica* to focus on conserving resources and minimizing root construction, while compensating with more efficient P absorption through changes in exudate characteristics, as we observed the highest increase in total organic acid secretion under P deficiency conditions (Figure 7c).

Early seedling growth depends on seed biomass and P reserves, which are typically depleted three weeks post-sowing [6]. If seedlings fail to obtain sufficient external P, growth will be hindered, potentially accelerating senescence. In such cases, P redistribution becomes essential for sustaining plant growth and enhancing tolerance to P deficiency [35]. In the present study, all three species allocated more P to their roots under P deficiency (Figure 3b). This reallocation supports plant growth by transferring P from less metabolically active tissues to those with higher metabolic activity. P reallocation promotes root development under low P conditions, thus improving P acquisition and utilization efficiency [36]. Additionally, our results showed that *L. bicolor* had the highest P concentrations in both leaf and stem tissue under P deficiency (*p* = 0.000 *) (Figure S1b). However, its total plant P content is lower than that of the other species (Figure 3b). This may be due to *L. bicolor* reallocating limited P to key tissues under P limitation. The increased P concentrations in the leaves and stem help maintain the function of these critical organs, which are essential for photosynthesis and growth, and may be a key factor in maintaining a higher RGR for *L. bicolor* under P deficiency.

Plant roots enhance P uptake through physiological plasticity, a key adaptive strategy for coping with P deficiency [37,38]. However, comprehensive research on P absorption kinetics in *Lespedeza* species, particularly inter-species comparisons, remains limited. In this study, we calculated key absorption kinetic parameters for P uptake, *I_max_*, *K_m_*, and *C_min_*, from P absorption curves for all three species. After 21 days of P-deficient culture, all three *Lespedeza* species exhibited significantly higher P absorption compared to those grown under P sufficiency, with the most significant increase observed in *L. bicolor* (Figure 4 and Figure 5). Similar findings have been reported in previous studies. For instance, *Brassica* demonstrated enhanced P absorption efficiency under P deficiency compared to sufficient P supply [23]. Additionally, maize grown at 0.2 μM P exhibited a 40-fold higher P uptake from the solution than that cultivated at 1 μM P [39]. These findings suggest that plants are capable of enhancing P absorption by adjusting root physiology in response to P deficiency. Previous studies have also demonstrated a relationship between absorption capacity and RGR, as P demand is satisfied by a continuously growing root, which results in a reduced demand per unit root length [40,41]. Our results confirmed this relationship, with *L. bicolor* showing the highest RGR and strongest P absorption capacity under P deficiency (Figure 2a and Figure 5).

In plant ecology, the *K_m_* and *I_max_* are key parameters for evaluating a plant’s adaptability to diverse nutritional environments. Cacco et al. [42] classified these parameters into four categories: (1) plants with low *K_m_* and high *I_max_* are capable of thriving in varied nutritional settings; (2) plants with high *K_m_* and high *I_max_* are adapted to rich environments; (3) plants with high *K_m_* and low *I_max_* tend to struggle in all environments; and (4) plants with low *K_m_* and low *I_max_* are adapted to low-nutrient conditions. In this study, *L. davurica* grown under P sufficiency showed low values for *I_max_* and *K_m_* (Figure 5), suggesting a tendency for adaptation to poor environments. This trait may have evolved gradually in response to the nutrient-deficient soils of semi-arid zones. Under P deficiency, *L. bicolor* exhibited the lowest *K_m_* and *C_min_* values, indicating its superior P absorption capability and excellent adaptability to low-nutrient environments [23,42]. Moreover, *L. bicolor* and *L. cuneata* showed more pronounced reductions in *K_m_* and *C_min_* values under P deficiency than *L. davurica*, reflecting potential differences in their adaptive strategies. This variation may be attributed to differences in root architecture and morphology: *L. bicolor* and *L. cuneata* possess more lateral roots, and their specific root length and area increased significantly under P deficiency, improving root contact with the nutrient solution (Figure 1c,d and Figure S2). In contrast, *L. davurica* features a prominent taproot with fewer lateral roots, and its root morphology did not show significant changes under P deficiency (Figure S2). However, the 21-day hydroponic experiment was conducted at 10 µM P, a concentration roughly 10 times higher than the typical < 1–2 µM found in natural soil solutions [21,22]. Consequently, the derived kinetic parameters (*K_m_*, *C_min_*) may overestimate the actual P-uptake capacity of these species under field conditions. In addition, a realistic appraisal of soil P availability must also account for P-buffering capacity and the slow reactions between phosphate and soil solids, as well as other soil chemical properties regulating P availability [43]. The low seed-P reserves coupled with relatively high whole-plant P content suggest that all three species could still absorb P at 10 µM (Table S1 and Figure S1), but longer-term studies under sustained low-P conditions—and preferably in real soil systems—are required to verify their steady-state *C_min_* and ultimate P-acquisition potential. 

The secretion of organic acids is a well-documented strategy that plants employ to tolerate P deficiency [24]. For example, guinea grass and palisade grass roots secrete malic acid and isocitric acid [44], while *Urochloa decumbens* and ryegrass release formic acid, acetic acid, and glycolic acid [45]. Additionally, P-efficient crops like canola and wheat secrete higher levels of malic acid and citric acid in low P conditions [46,47]. Although many studies report an increase in organic acid secretion under P deficiency, this response does not appear in all plant species [48]. Even within a single species, there are considerable variations in the types and levels of organic acids among different genotypes and cultivars [24]. In this study, we observed that under two different P treatments, the concentration of malic acid in the roots of three *Lespedeza* species was significantly higher than that of oxalic acid and citric acid (*p* = 0.000 *) (Figure 7a, b). Moreover, the increase in organic acid secretion was predominantly driven by an elevated secretion of malic acid (Figure 5). Prior to this study, little was known about organic acid secretion in *Lespedeza* species under P deficiency. Our findings indicate that malic acid is the predominant organic acid secreted by these species under P deficiency. This finding is consistent with observations in other leguminous species, such as Lupinus, where malic acid is the dominant organic acid anion secreted [24]. Interestingly, oxalic acid secretion has shown varying responses across different species under P deficiency. For instance, in soybean, oxalic acid secretion was significantly increased under P deficiency, suggesting a potential role in enhancing P uptake or tolerance [49]. In contrast, a study on ryegrass revealed a decrease in oxalic acid secretion when exposed to P deficiency, indicating that the response may be species-specific [50]. In this study, oxalic acid secretion was significantly increased under P deficiency. Additionally, the significant concentration difference between malic acid and citric acid may be attributed to rhizosphere pH, as Yang et al. [51] demonstrated that pH is a key factor influencing the relative ratio of malic acid to citric acid. However, it is important to note that the sampling process could have caused the roots to be intermittently exposed to light, which may influence their metabolic activity and subsequent organic acid secretion. Moreover, the 3 h sampling window might not fully capture the natural dynamics of organic acid exudation. These conditions might introduce biases, as they differ from the actual rhizosphere environment, where roots in the sampling process are frequently exposed to changes in light and nutrient availability. Furthermore, variations in organic acid exudation are closely linked to factors such as cultivation time, photosynthetic duration, and the development of cluster roots [15,31,52].

In addition to organic acid secretion, phosphatases play a crucial role in the acquisition of inorganic phosphate (Pi) in the plant rhizosphere [53,54,55]. Acid phosphatases, commonly found in root exudates, hydrolyze organic phosphate to release Pi [38,39]. Numerous studies have demonstrated that acid phosphatase activity increases under P deficiency [56,57]. One study reported that under P deficiency, acid phosphatase activity hotspots in lupins were 40% larger than those under P sufficiency [58]. Consistent with these findings, our study revealed significantly higher acid phosphatase activity of the three *Lespedeza* species under P deficiency, with the most pronounced increase in *L. bicolor* (*p* = 0.000 *) (Figure 6). Therefore, increased acid phosphatase activity appears to be a common strategy employed by these species to cope with P deficiency. However, phosphatase assays only reflect potential activity, and various factors such as plant developmental stage, soil type, pH, and temperature can influence the results [59,60]. More systematic research is needed to examine the differential responses of phosphatase activity among the three *Lespedeza* species.

## 4. Materials and Methods

### 4.1. Plant Materials, Growth Conditions, and Phosphate Supplementation

Seeds of *L. davurica* were collected from wild populations in Yan’an, Shaanxi (109°32′85′′ E, 36°86′32′′ N). Seeds of *L. bicolor* were purchased from Beijing Zhengdao Seed Industry Co., Ltd., and seeds of *L. cuneata* were sourced from Ningxia Shanggu Agricultural and Livestock Development Co., Ltd (Yinchuan, China). All seeds were surface-sterilized using a 15% sodium hypochlorite solution for 15 min. After sterilization, the seeds were sown in plastic pots filled with a vermiculite-perlite mixture (*v*:*v* = 1:1) for an initial 10-day growth period. Following this, 15 seedlings from each species were transferred to a foam board for hydroponic cultivation in opaque plastic pots containing 6 L of nutrient solution (pH 6.0). A total of six pots were used per species, with three pots assigned to each of the two treatments, and each pot treated as a biological replicate, resulting in 18 pots in total.

The experiment was conducted using two different P levels: 0.01 mM NaH_2_PO_4_ (P-deficiency, −P) and 0.2 mM NaH_2_PO_4_ (P-sufficiency, +P). The P concentration was the only difference between the two treatments, while the other components of the nutrient solution were identical for both, with the following components: K_2_SO_4_ (2 mM), NH_4_NO_3_ (2.1 mM), MgSO_4_ (2 mM), CaCl_2_ (1.2 mM), EDTA-FeNa (0.1 mM), H_3_BO_3_ (0.01 mM), ZnSO_4_ (0.001 mM), MnSO_4_ (0.001 mM), CuSO_4_ (0.2 μM), and (NH_4_)_6_Mo_7_O_24_ (0.005 μM). Hydroponic cultivation was conducted for three weeks. During the first two weeks, the nutrient solution was replaced every three days, and during the final week, it was replaced every two days. Both seed germination and hydroponic cultivation were performed in a temperature-controlled growth chamber with light and dark cycles of 14 h (25 °C) and 10 h (22 °C), respectively.

### 4.2. Morphological Parameters and Growth Analyses

Seven days after transplantation (DAT), five seedlings per treatment were collected to evaluate the relative growth rate (RGR). Roots, stems, and leaves were dried at 80 °C until a constant weight was reached, and their dry weights were recorded. At 21 DAT, five seedlings per treatment were again collected, and their roots and leaves were scanned using an Epson Perfection V800 Photo scanner (Epson America Inc., Hillsboro, OR, USA). The samples were subsequently dried to determine the dry weights of the roots, stems, and leaves. The leaf projected area, total root length, root volume, and root surface area were quantified from scanned images using WinRHIZO 2017 Operator (Regent Instruments Inc., Quebec, QC, Canada).

Specific root area, volume, and length were calculated as the ratios of root surface area, volume, and total length to root dry weight at 21 DAT. RGR was calculated based on the changes in dry weight between 7 DAT and 21 DAT, normalized to the dry weight at 21 DAT. The net assimilation rate (NAR) and leaf weight ratio (LWR) were calculated using the following formulas [24,61]:

(1) $$\mathrm{RGR}\;=\;\frac{DW}{DT}\times \;\frac{1}{W}=\;\frac{1}{LA}\times \;\frac{DW}{DT}\times \;\frac{LA}{W}\;=\;LAR\times \;NAR$$

(2) $$\mathrm{LAR}=\frac{LW}{W}\times \;\frac{LA}{LW}\;=\;SLA\times \;LWR$$

In the formulas, W represents the dry weight of the entire plant, LA is the projected leaf area, LW is the leaf dry weight, LAR is the leaf area ratio, and DW represents the change in dry weight between plants harvested at 21 DAT and 7 DAT.

### 4.3. Root Exudate Collection

At 21 DAT, one plant from each of the three pots per treatment was randomly selected for root exudate collection. The roots were immersed in a glass container with 200 mL of 0.05 mM CaCl_2_ solution for 3 h. This process was conducted in an artificial climate chamber, where the plant shoots were exposed to light, and the roots were kept in the dark. Acid phosphatase activity was measured immediately after exudate collection, while the samples for organic acid analysis were stored at −20 °C.

### 4.4. Measurement of Organic Acids and Acid Phosphatase Activity

Prior to the organic acid assay, 10 mL of the collected solution was freeze-dried to a final volume of 1 mL and then filtered through a 0.45 µm membrane. The organic acid content was determined using the Shimadzu LC-20A Prominence UFLC System (Shimadzu Corporation, Kyoto, Japan). Separation of the organic acids (oxalic, malic, and citric acid) was performed on a SHIMADZU Shim-pack VP-ODS-C_18_ column (4.6 × 150 mm, 5 µm; Shimadzu Corporation, Kyoto, Japan). The detection conditions were as follows: column temperature set at 30 °C; mobile phase consisting of acetonitrile (Aladdin, Shanghai, China) and 0.01 M KH_2_PO_4_ (pH 2.50); flow rate of 0.6 mL/min; and injection volume of 10 μL. Detection wavelengths were programmed according to Zhang et al. [62]. Total organic acids were calculated as the sum of oxalic, malic, and citric acid concentrations.

For the root-released acid phosphatase activity assay, 0.5 mL of the collected solution was mixed with an equal volume of 10 mM p-nitrophenyl phosphate (pNPP, Aladdin, Shanghai, China) (dissolved in 0.2 M acetate buffer, pH 5.5). The mixture was incubated at 30 °C for 30 min, and the reaction was terminated with 0.2 M NaOH. The p-nitrophenol (pNP) produced was quantified by measuring the optical density at 400 nm. Acid phosphatase activity was expressed as the amount of pNP generated per unit root fresh weight per unit time.

Each treatment group included three biological replicates, and the measurements were performed independently for each replicate to ensure statistical reliability.

### 4.5. Absorption Kinetics Test

The absorption kinetics test was conducted using a previously reported depletion method [63]. At 21 DAT, one plant from each of the three pots per treatment was randomly selected for the test. Initially, seedlings were subjected to nutrient starvation in a 0.5 mM CaCl_2_ solution for 48 h to eliminate the effects of residual P contained in the apoplast [64]. The seedlings were then transferred to glass bottles containing 200 mL of nutrient solution with 0.1 mM P. Samples (2 mL) were collected from each bottle at 0.5, 1, 1.5, 2, 3, 5, and 8 h post-transfer, with each sample immediately replaced by 2 mL of deionized water. The P concentration in the samples was analyzed to assess the P absorption dynamics among the three *Lespedeza* species. Each treatment group included three biological replicates.

A second-order polynomial was fitted to the P concentration versus absorption time data to generate P consumption curves and calculate the absorption kinetics parameters [24].

Y = cX^2^ + bX + a (3)

In the equation, Y represents the P concentration, and X denotes the absorption time.

The change in the concentration rate with absorbing time was calculated using the following formula:

Y′ = −(b + 2cX)(4)

The maximum change rate in P concentration occurs when X = 0, which results in Y′ = −b. The maximum potential for plant P absorption (*I_max_*) was calculated using the following formula:

(5) $$I_{\max}\;=\;-b\;\frac{V}{m}$$

In the formula, m represents the root dry weight, and v represents the volume of the solution. When the absorption rate is half of the *I_max_*, the corresponding P concentration in the solution is equal to the Michaelis constant (*K_m_*). The value 1/*K_m_* reflects the affinity of the roots of the three *Lespedeza* species for P. When the absorption rate is 0, the P concentration in the solution corresponds to the equilibrium concentration (*C*_min_).

### 4.6. Phosphorus Measurement

The P concentration in the nutrient solution was determined using the vanadomolybdate blue method [65], which involves the formation of a yellow vanadomolybdate complex in the presence of phosphate ions, which can be reduced to a blue-colored complex, and the intensity of the blue color is proportional to the P concentration. To assess the P content in the roots, stems, and leaves, plants were collected at 21 DAT and digested in H_2_SO_4_-H_2_O_2_ solution. The P content in the digested samples was subsequently analyzed using the same method. Each treatment group included three biological replicates, and the measurements were performed independently for each replicate.

### 4.7. Statistical Analysis

The biomass and morphology were tested in quintuplicate, and the remaining tests were performed in triplicate. The statistical analyses were performed using SPSS 22.0 (SPSS Inc., Chicago, IL, USA). A one-way analysis of variance (ANOVA) was used to evaluate the differences among the three *Lespedeza* species and two P treatments. Post hoc comparisons of means were conducted using Duncan’s multiple range test, with statistical significance set at *p* < 0.05. The results are presented as mean values ± standard deviation (SD). All graphs were generated using Origin 2022 software (OriginLab Corporation, Northampton, MA, USA).

## 5. Conclusions

This study highlights the distinct strategies employed by three *Lespedeza* species—*L. davurica*, *L. bicolor*, and *L. cuneata*—to cope with P deficiency. *L. bicolor* demonstrated the greatest tolerance to P deficiency, showing minimal reduction in RGR and higher P content in stems and leaves compared to the other two species. In contrast, *L. davurica* and *L. cuneata* allocated more carbon to root growth in response to P deficiency, with both species exhibiting enhanced organic acid secretion. However, *L. bicolor* stood out for its superior P absorption efficiency and higher acid phosphatase activity, suggesting a more effective mechanism for P acquisition. Overall, these findings underscore the variability in P response strategies across *Lespedeza* species, with *L. bicolor* emerging as the most adaptable to P-limited environments. However, these results are based on the performance of the three species under hydroponic conditions at 21 DAT with two P levels (0.01 vs. 0.2 mM). Future studies should verify these mechanisms in field conditions, considering soil, microbiome, time scale, and other factors, and further investigate the molecular basis of these adaptive traits to enhance P utilization in these plants.

## Data Availability

Data will be made available on request.

## Supplementary Materials

The following supporting information can be downloaded at https://www.mdpi.com/article/10.3390/plants14203124/s1: Figure S1: The P concentration (a, b) of leaves, stems, roots, and P use efficiency (c) of three *Lespedeza* species under different P levels; Figure S2: Seeds of three *Lespedeza* species; Figure S3: The different root morphologies of three *Lespedeza* species at 21 DAT under different phosphorus treatments; Table S1: The hundred-grain weight, P concentration, and P content of three *Lespedeza* species; Table S2: Information on chemicals used in this study.
