# Supplementary material for: Differential Phosphorus Acquisition Strategies and Adaptive Mechanisms Evolved by Three Lespedeza Species to Tackle Phosphorus Deficiency
Source: Plants (Basel). 2025 Oct 10;14(20):3124. doi: 10.3390/plants14203124 (PMC12566934; doi:10.3390/plants14203124)
Supplement: Supplementary file 1 [file plants-14-03124-s001.zip › plants-3859561-supplementary.pdf]

Table S1 The hundred-grain weight, P concentration, and P content of three *Lespedeza* species.

Species	Hundred-grain weight (g)	P concentration (mg P g^{-1})	P content (μg P $seed^{-1}$)
L. davurica	0.204 ± 0.008	2.82 ± 0.06	5.75 ± 0.12
L. bicolor	0.732 ± 0.006	2.48 ± 0.10	18.15 ± 0.68
L. cuneata	0.209 ± 0.004	2.33 ± 0.09	4.87 ± 0.09

Table S2 Information on chemicals used in this study.

Chemical Name	Chemical Formula	Source	Manufacturer Location & Country
Potassium sulfate	K_2SO_4	Solarbio	Beijing, China
Magnesium Sulfate	$MgSO_4$	Solarbio	Beijing, China
EDTA-FeNa	EDTA-FeNa	Solarbio	Beijing, China
Boric acid	H_3BO_3	Solarbio	Beijing, China
Ammonium nitrate	NH_4NO_3	Tianjin Kemio	Tianjin, China
Calcium chloride	$CaCl_2$	Tianjin Kemio	Tianjin, China
Zinc sulfate	$ZnSO_4$	Tianjin Kemio	Tianjin, China
Ammonium heptamolybdate	$(NH_4)_6Mo_7O_{24}$	Tianjin Kemio	Tianjin, China
Potassium dihydrogen phosphate	KH_2PO_4	Tianjin Kemio	Tianjin, China
Sodium hydroxide	NaOH	Tianjin Kemio	Tianjin, China
Sulfuric acid	H_2SO_4	Tianjin Kemio	Tianjin, China
Hydrogen peroxide	H_2O_2	Tianjin Kemio	Tianjin, China
Calcium chloride	$CaCl_2$	Tianjin Kemio	Tianjin, China
Manganese sulfate	$MnSO_4$	Aladdin	Shanghai, China
Acetonitrile	CH_3CN	Aladdin	Shanghai, China
p-Nitrophenyl phosphate	p-nitrophenyl phosphate	Aladdin	Shanghai, China

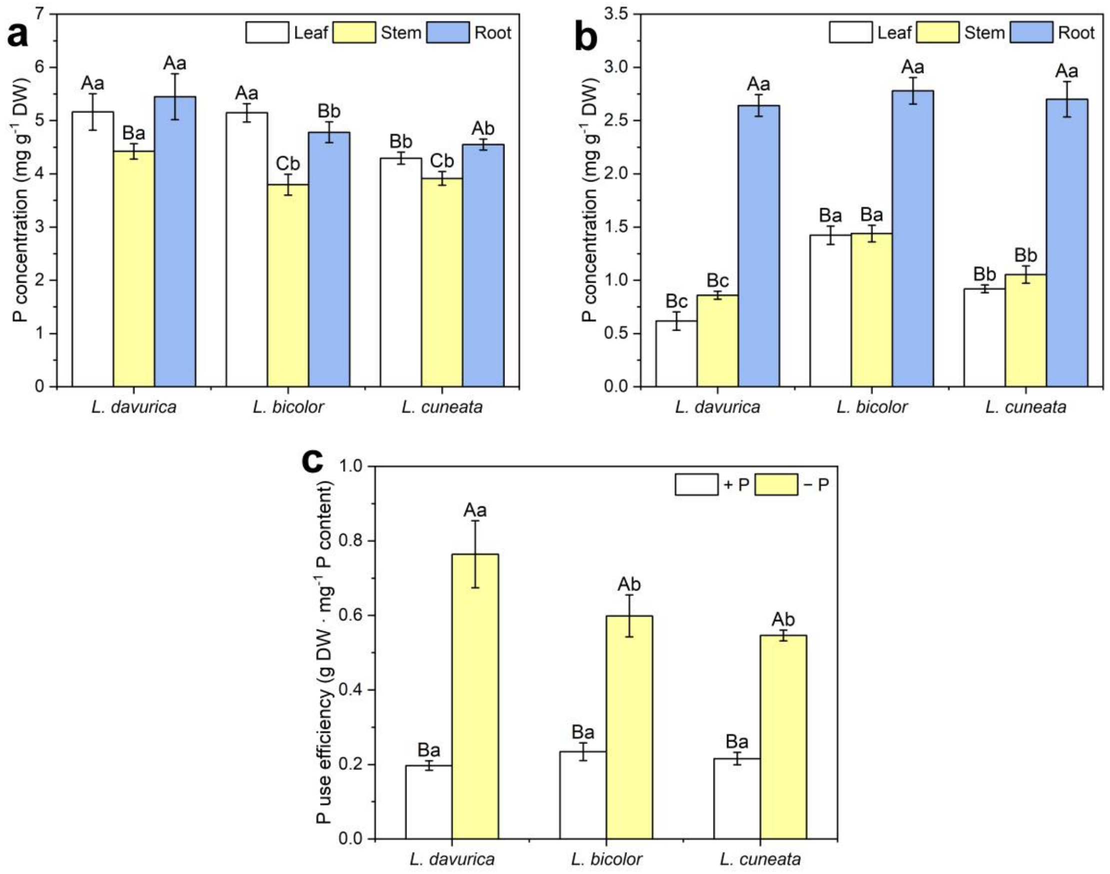

Figure S1 The P concentration **(a, b)** of leaves, stems, roots, and P use efficiency **(c)** of three *Lespedeza* species under different P levels. a, P-sufficient; b, P-deficient. Data are shown as Mean ± SD (n = 3). One-way ANOVA was performed to compare differences in organic acid content within the same species at the same P level, differences between species for the same organic acid, as well as differences in total organic acid content within the same species at different P levels and between species at the same P level, followed by post-hoc Duncan's multiple range test. In figures **(a)** and **(b)**, different uppercase letters indicate significant differences in organic acid content within the same species at the same P level ($p < 0.05$), and different lowercase letters indicate significant differences between species for the same organic acid at the same P level ($p < 0.05$). In the figure **(c)**, uppercase letters indicate significant differences in total organic acid content within the same species at different P levels ($p < 0.05$), and lowercase.

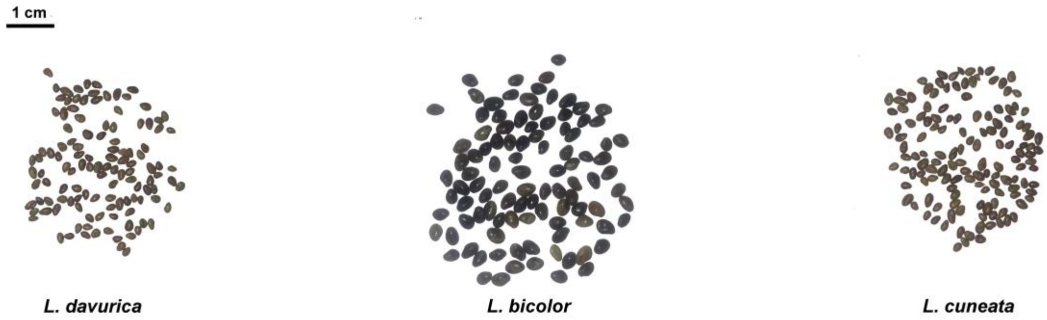

Figure S2 Seeds of three *Lespedeza* species.

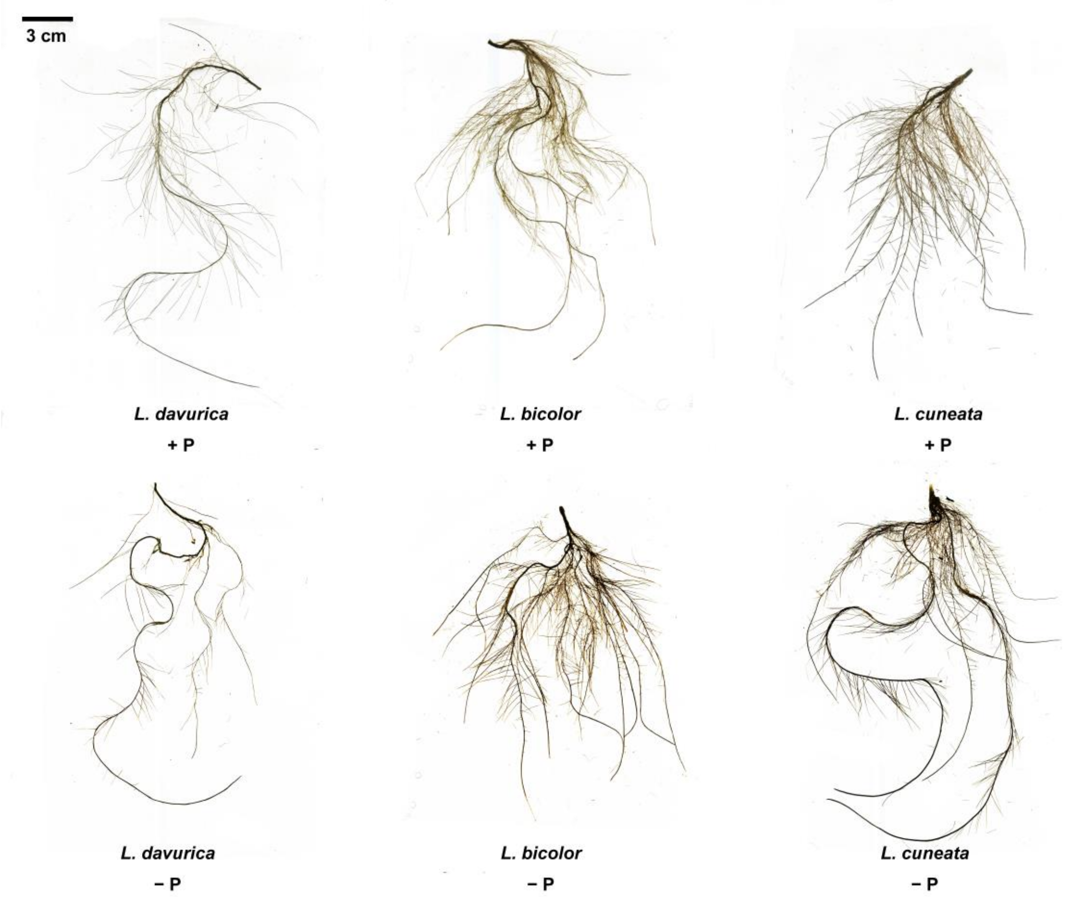

Figure S3 The different root morphologies of three *Lespedeza* species at 21 DAT under different phosphorus treatments.
